# Combined treatment of somatostatin analogues with pegvisomant in acromegaly

**DOI:** 10.1007/s12020-015-0810-8

**Published:** 2015-12-10

**Authors:** S. E. Franck, A. Muhammad, A. J. van der Lely, S. J. C. M. M. Neggers

**Affiliations:** Department of Internal Medicine, Endocrinology Section, Pituitary Center Rotterdam, Erasmus University Medical Center, PO Box 2040, 3000 CA Rotterdam, The Netherlands

**Keywords:** Acromegaly, Somatostatin analogues, Growth hormone receptor antagonist, Pegvisomant, Growth hormone, Insulin-like growth factor I

## Abstract

Treatment of acromegaly with monotherapy long-acting somatostatin analogues (LA-SSA) as primary treatment or after neurosurgery can only achieve complete normalization of insulin-like growth factor I (IGF-I) in roughly 40 % of patients. Recently, one of the acromegaly consensus groups has recommended switching to combined treatment of LA-SSA and pegvisomant (PEGV) in patients with partial response to LA-SSAs. This combination of LA-SSA and PEGV, a growth hormone receptor antagonist, can normalize IGF-I levels in virtually all patients, requiring that the adequate dose of PEGV is used. The required PEGV dose varies significantly between individual acromegaly patients. One of the advantages of the combination therapy is that tumor size control or even tumor shrinkage can be observed in a vast majority of patients. The main side effects of the combination treatment are gastrointestinal symptoms, lipohypertrophy and transient elevated liver transaminases. In this review we provide an overview of the efficacy and safety of the combined treatment of LA-SSAs with PEGV.

## Introduction

Acromegaly is a rare disease almost exclusively caused by a growth hormone (GH) secreting pituitary adenoma. This hypersecretion of GH and the associated elevated IGF-I levels result in increased morbidity and mortality [1]. Surgery is in general the first treatment modality, but is not always successful, as the majority of the patients have a macroadenoma [1]. The reported cure rates of transsphenoidal surgery vary widely, mainly depending on tumor size and invasiveness and the experience of the neurosurgeon [2]. Surgery of microadenomas (<1 cm in diameter) has an average cure rate of 78 %, whereas with macroadenomas (≥1 cm in diameter) the average cure rate ≤50 % [2]. However, these previous results were obtained 10 years ago. More recent data from daily practice were shown in a study with data gathered from the UK National Acromegaly Registry. This study reported cure rates between 20 and 40 % [3]. LA-SSAs are currently used as pre-surgical treatment, adjuvant treatment and as primary medical treatment. Multiple studies addressed the efficacy of LA-SSA, and have shown that LA-SSA treatment alone reached normalization of IGF-I levels in about half of the patients [4]. No differences between lanreotide SR and octreotide LAR were observed regarding tumor shrinkage and normalization of IGF-I levels [5]. An attractive way to control biochemical disease activity in patients that are uncontrolled on LA-SSA monotherapy is to add PEGV, because of the different modes of action of these two drugs. LA-SSA reduces hypersecretion of GH by binding to somatostatin receptors on the pituitary adenoma. PEGV is a pegylated recombinant analogue of GH and thereby functions as a GH antagonist. It acts by reducing the excessive GH actions in peripheral tissues and by blocking the increased production of IGF-I by the liver. Combined treatment as pre-surgical treatment to improve morbidity is very questionable, as there are yet no supporting data. Adjuvant treatment and primary medical treatment are suitable for LA-SSA combined with PEGV. However, in the current guidelines combination treatment is only recommended in partial responders to LA-SSA [6]. A recent Italian registration study reported that LA-SSA combined with PEGV is prescribed widely in Italy and more frequently in tertiary than in secondary referral hospitals (59 vs. 37 % of the patients, *p* < 0.001). In more experienced centers less adverse events were reported during PEGV treatment, however long-term efficacy rates were lower. The authors suggest for this latter finding an inadequate patient’s selection due to prevalence of more aggressive tumors in the tertiary referral centers.

The first clinical PEGV-trials observed high efficacy rates of 89–97 % depending on dose and duration of the treatment [7, 8]. The large observational ACROSTUDY™ could not confirm these high efficacy rates. However, it is noteworthy that this non-interventional safety surveillance study was not designed to address efficacy rates, as dose titration was not part of the study design [9]. The first study about combined treatment with both LA-SSA and PEGV was reported in 2005, which observed a high efficacy rate of 95 % with a lower required PEGV dose to achieve normalization of IGF-I levels compared to the first data reporting PEGV monotherapy with a similar efficacy rate [8, 10]. This high efficacy rate and lower required PEGV dose during combined treatment was confirmed in later reports [11–14]. This review will discuss the available literature on the combination treatment in acromegaly patients, including level of disease control, tumor size and side effects.

## Efficacy of the combination therapy with LA-SSA and PEGV

**Fig. 1:**
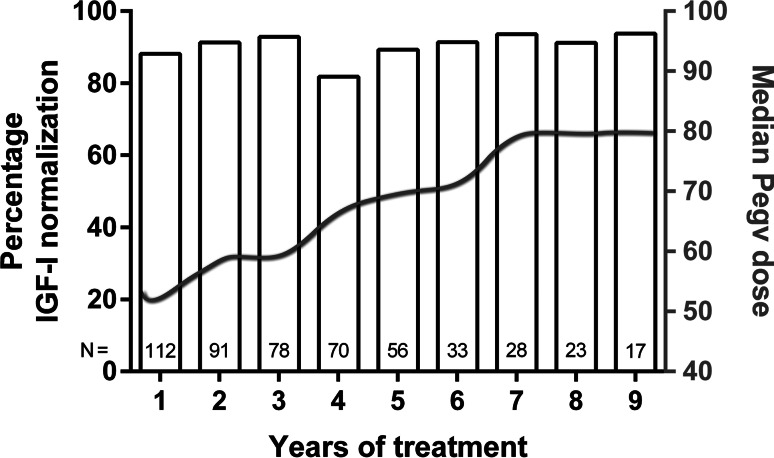
Efficacy of combination treatment. Percentages of patients with IGF-I < 1.2 ×ULN and median PEGV doses (*grey line, right Y-*axis) are shown for every individual year during 9 years of combination treatment, LA-SSA combined with PEGV. Median PEGV dose is in mg weekly. Cumulative numbers of the included patients at each treatment year are depicted at the bottom of every bar. All patients (*n* = 112) were treated for at least 1 year, 17 patients were treated for a maximum of 9 years of follow up. LA-SSA: Long-acting somatostatin analogues; *PEGV* pegvisomant, *IGF*-*I* insulin like growth factor I. This figure was reproduced with permission from [12]

Meta-analyses of clinical trials showed that LA-SSAs alone normalize GH and IGF-I levels in about 50 % of patients [4]. However, due to selection bias this efficacy rate is probably an overestimation. In unselected treatment-naive patients an LA-SSA efficacy rate of 40 % seems to be more common [15, 16]. PEGV appears to be more effective, both during monotherapy as during co-treatment with LA-SSA [7, 8, 11–14, 17]. Table 1 shows a summary of studies reporting on efficacy of acromegaly patients using LA-SSA in combination with PEGV. The efficacy rates during a period of 9 years in a large Dutch cohort are shown in Fig. 1 [12]. Because PEGV is a competitive blocker of the GH receptor (GHR), pharmacology dictates that in principle it should be possible to control IGF-I levels in all patients with acromegaly, provided that the appropriate PEGV dose is used. LA-SSAs have direct and indirect effects that result in a GH-independent decrease of IGF-I production [18, 19]. A Danish study reported that PEGV serum levels increase by 20 % when combined with LA-SSA [20]. The appropriate PEGV dose varies among acromegaly patients, the Dutch cohort reported that patients using high dose of LA-SSA needed a median weekly PEGV dose of 80 mg (range: 30–300 mg) to achieve normal IGF-I levels in 97 % of the patients [12]. Monotherapy of PEGV requires a higher cumulative weekly dose of around 130 mg to achieve a similar normalization rate after 12 months of treatment [8]. In contrast to these reports, an Italian observational study reported no difference in median required PEGV dose to normalize IGF-I levels in patients using PEGV monotherapy compared to patients treated with the combination therapy [21]. However, these groups were not similar according to severity of the disease. The PEGV monotherapy group had significant lower GH and IGF-I levels at baseline compared to the combination group in this Italian observational study [21]. Observational registries such as the ACROSTUDY™ and German Pegvisomant Observational Study (GPOS) [9, 22], in which patients using PEGV are included regardless their concomitant medication, observed lower efficacy rates around 60 %, compared with the clinical trials of PEGV monotherapy and combination treatment mentioned previously [8, 12]. In the ACROSTUDY™ PEGV was combined with LA-SSA in 23 %, with dopamine agonist in 6 %, and a combination of the three agents in 4 % of patients [9]. After 5 years the mean weekly dose was 106 mg in patients with a normal IGF-I, and 113 mg in those with an elevated IGF-I [23]. However, these studies were not designed to evaluate efficacy and dose titration, but aimed for the evaluation of safety aspects, such as rare side effects. The lower efficacy rates in the observational registration studies might be explained by the relative lower dose of PEGV. To achieve efficacy rates of more than 90 % during PEGV monotherapy, the average expected weekly dose is probably above 120–130 mg.

**Table 1:** Summary of studies reporting on combination treatment

First author, year, (Ref)	Design	Aim of study	No. of patients	Disease control (%)	PEGV dose (mg/weekly)	Duration study (months)
Van der Lely et al. [24]	Case report	IGF-I normalization	1	100	280	18
Trainer et al. [17]	Randomized controlled trial	Primary end-point: AEs, secondary end-point: IGF-I normalization	29	73	105	9
Van der Lely et al. [14]	Prospective observational study	IGF-I normalization and AEs	57	79	60	7
Bianchi et al. [21]	Retrospective observational study	IGF-I normalization and AEs	27	67	140	30 (median)
Neggers et al. [12]	Retrospective observational study	IGF-I normalization and AEs	112	97	80	59 (median)

Summary of studies reporting on LA-SSA combined with PEGV and the percentage of disease control (normalization of IGF-I levels) and the required PEGV dose in order to control IGF-I levels. The ACROSTUDY is not included in this table as it includes patients with monotherapy of PEGV and various other medical combinations with PEGV

*LA-SSA* long-acting somatostatin analogues, *PEGV* pegvisomant, *IGF-I* insulin like growth factor I, *AEs* adverse events

Escape, defined as need to increase the dose of PEGV because of an increase in IGF-I levels, was reported in 34 % of a Spanish cohort [25]. The majority of patients were easily controlled with either an increase in PEGV dose, additional medical treatment or both. Whether this increase in the necessary dose of PEGV should be called an escape is questionable, as most patients were easily controlled and remained controlled on a higher dose of PEGV. A significant number of these patients escaped from PEGV within the first 6 months after discontinuation of LA-SSA. Presumably during this period these patients were actually still receiving combination treatment, due to the long half-life of LA-SSAs. The Dutch cohort could not observe this phenomenon during combination treatment in acromegaly patients [12].

Co-administration of the highest dose of LA-SSA on top of PEGV monotherapy appears to reduce the necessary mean PEGV dose by 50 % [14], although with a high individual variability [26]. An analysis including eight patients whose mean IGF-I levels were similar during PEGV monotherapy, showed that these patients were able to reduce their PEGV dose from 131.3 ± 36.2 to 62.5 ± 16.7  mg weekly [14]. PEGV dose reduction during combination treatment might improve cost-effectiveness of medical treatment in acromegaly and may reduce injection frequency for patients. However, there are no direct studies comparing cost-effectiveness of the median required weekly PEGV dose during mono- and combination treatment.

## Improved quality of life by the addition of PEGV

Physicians tend to be mainly focused on biochemical parameters as GH and IGF-I levels during treatment of acromegaly. These parameters are definitely linked to a better outcome and a lower risk of morbidity and mortality [27, 28]. A recent French study has described long-term effects of PEGV on cardiorespiratory and metabolic comorbidities [29]. They reported that long-term PEGV treatment improves cardiac function measured by the left ventricular ejection fraction and the left ventricular mass index. PEGV also improved the apnea–hypopnea index. Normalized serum GH and IGF-I levels does not necessarily result in complete resolution of signs and symptoms [30, 31]. Neggers et al. reported on the results of a prospective, double blind, placebo controlled, crossover study, and demonstrated improved quality of life (QoL) in patients using LA-SSA combined with low-dose PEGV [31]. QoL was assessed by two acromegaly specific QoL-questionnaires, the AcroQoL and the PASQ. Improved QoL was observed without significant changes in IGF-I levels after addition of PEGV to LA-SSA therapy in patients with normalized IGF-I levels during monotherapy of LA-SSA. However, a study by Madsen et al. did not observe an improved QoL in patients using LA-SSA combined with a low dose of PEGV [32]. Two differences in the design of the studies might clarify these different outcomes in QoL; (1) Madsen et al. reduced the dose of LA-SSA by 50 % before adding low-dose PEGV treatment, while Neggers et al. did not change the LA-SSA dose; (2) disease specific QoL-questionnaires were used by Neggers et al. whereas Madsen et al. used QoL-questionnaires which were not disease specific.

An explanation for the observed improvement in QoL by the addition of low-dose PEGV could be a persistent systemic acromegaly disease activity during monotherapy of LA-SSA that has been hypothesized and was called ‘extra-hepatic acromegaly’ [33]. LA-SSA treatment selectively decreases hepatic-IGF-I production by around 20 % via direct and indirect mechanisms [18, 19, 34]. This might lead to an overestimation of the efficacy of LA-SSAs to normalize IGF-I via a reduction in the pathological GH secretion because of this GH-independent reduction of IGF-I levels. Therefore, GH actions on extra-hepatic tissues can remain elevated despite normalization of serum IGF-I levels. LA-SSA reduces portal insulin levels which decreases hepatic GHR expression only [34]. This results in a relatively hyper-GH-sensitive state of the other tissues. Patients report these still excessive GH actions on non-hepatic tissues such as edema, fatigue and headaches comparable to the side-effects of high-dose GH replacement therapy in GH deficient subjects. A clinical example is a Danish study which observed, despite similarly normalized IGF-I levels, that LA-SSA treatment compared with neurosurgery alone was associated with less suppressed GH levels and less symptom relief [35]. Blocking non-hepatic GH actions by low-dose PEGV could therefore be useful in treating this ‘extra-hepatic acromegaly’ [33]. Moreover, PEGV has also been shown to improve insulin resistance by several mechanisms [36–41], which is beneficial in the presence of LA-SSAs, which are known to reduce insulin secretion [42, 43].

## Pituitary tumor volume

Recent prospective multicenter clinical trial observed tumor shrinkage in 63 % of primary treated patients with 120 mg Lanreotide Autogel administered every 28 days [44]. Tumor shrinkage was defined as clinically significant when ≥20 % tumor volume reduction was observed after 48 weeks of Lanreotide Autogel administration. While LA-SSA is successful in reducing tumor volume, previous concerns were raised whether PEGV might induce growth of the pituitary adenoma. Despite the fact that in a few cases an increase in tumor size during PEGV therapy was reported, there are no data suggesting that PEGV directly promotes tumor growth [14, 45, 46]. In the GPOS study, changes in tumor size were systematically monitored in 307 patients, from which 28 patients were treated with PEGV in combination with LA-SSA, however predominantly treated with PEGV monotherapy [47]. In eight out of 307 (2.7 %) an initial increase in tumor size was reported. In only three of these eight patients a real, but minor, increase in tumor size after PEGV treatment was observed [47]. In two of these patients a detectable rebound increase in tumor size after discontinuation of LA-SSA therapy was the probable reason of this increase in adenoma size. In a Spanish study in 75 patients, 5 (6.7 %) acromegaly patients were identified with an increase in pituitary tumor size [48]. All of these patients were pre-treated with LA-SSA and then switched to PEGV monotherapy. Noteworthy in this study is that the reference MRI was made just after LA-SSA was discontinued [48]. Therefore, the reported tumor size increases in this study may also be explained by the rebound phenomenon after cessation of LA-SSA treatment. In the Dutch cohort of acromegaly patients (*n* = 141) on combination therapy, growth of the adenoma has been reported in only one patient, while this growth was already observed before the addition of PEGV [12]. Moreover, during combined treatment tumor size shrinkage of more than 20 % of the largest diameter before and during combination treatment was observed in 17 % of the patients and the vast majority had a stable tumor volume. We, therefore, conclude that PEGV apparently does not influence the natural course of tumor growth, but ongoing alertness is required to monitor tumor size by repetitive pituitary imaging.

## Side effects

PEGV treatment is generally well tolerated and is increasingly considered to be safe [9, 49]. As LA-SSAs are on the market since 1988, more data are available on side effects. Most commonly reported are gastrointestinal symptoms such as nausea, vomiting, abdominal pain, biliary sludge or gallstones and diarrhea [50]. Abnormal glucose metabolism and injection-site reactions are also described [50]. Lipohypertrophy and hepatotoxicity are the most frequently reported adverse events during long-term PEGV treatment. Lipohypertrophy is an increased subcutaneous fat deposition around the injection sites of PEGV. It is suggested that the blockade of GH receptors causes unopposed insulin effects and, therefore, promotes lipogenesis [51]. The symptom arises in 3–15 % of the PEGV users either during monotherapy or combination treatment and appeared to be reversible by a more frequent rotation of the injection-site [12, 14, 21, 25, 52]. However, in one of the Dutch patients during combined treatment cosmetic surgery was necessary in order to reduce the significant lipohypertrophy [12]. Large observational studies as ACROSTUDY™ and GPOS, in which patients using PEGV are included regardless their concomitant medication, reported 2.2 and 7.4 % lipohypertrophy respectively.

Hepatotoxicity can occur during both PEGV monotherapy and combination therapy. Cholestasis is a common side effect of LA-SSA, as it reduces the secretion of cholecystokinin and motility of the gallbladder and induces an increase in bile concentration. This results in an increased risk in development of sludge and gallstones [53, 54]. LA-SSA often induces asymptomatic cholelithiasis, but an acute cholecystitis has been observed in only a minority of these patients [55]. The PEGV-induced elevations in hepatocellular enzymes are usually mild and self-limiting, both during monotherapy PEGV as in combination with LA-SSA [21, 52]. Transient elevated transaminases (TET) of more than three times the upper limit of normal (>3 ×ULN) were observed in 11.3–13.5 % of the patients using LA-SSA combined with PEGV [12, 21]. One Italian study compared long-term treatment of PEGV alone with PEGV in combination with LA-SSA regarding to TET [21]. Incidence of TET during monotherapy of PEGV was reported in 14.3 % of the patients, while this incidence was 11.1 % in the combined group. In the Dutchcohort (*N* = 19/141) all cases were transient without PEGV dose adaptation or discontinuation of the drug, except for one patient [12]. More details about TET patients are shown in Table 2. The development of TET was not PEGV dose-dependent. The ACROSTUDY™ and GPOS reported lower incidence rates of TET >3 ×ULN, 2.5 and 5.2 % respectively. The most recent ACROSTUDY™ paper, the Italian experience as it includes only patients registered in Italy (*n* = 341), reported an increase of TET >5 ×ULN in 0.9 % of the patients after 3 months of PEGV use and TET normalized after PEGV was withdrawn.

**Table 2:** Transient elevated transaminases during combination treatment

	Sex	Age	Time between start PEGV and TET (months)	PEGV dose during TET	Peak LFT (×ULN)					Follow up
					Bili	Alk. Phos.	*γ*-GT	AST	ALT	
Biering et al. [56]										
1	M	43	2.3	10 mg daily	NA	NA	NA	3.1	4.9	Drug withdrawn, norm. of TET
2^a^	F	44	2.2	15 mg daily	NA	NA	NA	11.1	20.8	Drug withdrawn, norm. of TET
Soto Moreno et al. [57]										
1	F	31	1.5	10 mg daily	0.3	0.3	1.4	117	80	Drug continued, norm. of TET
Neggers et al. [12]										
1	M	41	61.9	30 mg weekly	0.7	0.6	2.0	4.0	4.7	Drug continued, norm. of TET
2	M	39	19.5	300 mg weekly	NA	1.0	1.8	5.0	5.5	Drug continued, norm. of TET
3	M	60	2.7	40 mg weekly	0.4	1.2	0.9	4.3	7.0	Drug continued, norm. of TET
4	M	60	58.3	160 mg weekly	0.4	1.2	1.8	4.6	6.5	Drug continued, norm. of TET
5	M	45	3.2	80 mg weekly	1.1	0.5	2.0	3.5	3.9	Drug continued, norm. of TET
6^a^	M	51	3.9	60 mg weekly	2.3	2.9	16.7	16.7	25.8	Drug continued, norm. of TET
7	M	59	3.5	60 mg weekly	0.6	0.8	1.8	2.4	3.6	Drug continued, norm. of TET
8^a^	M	29	18.8	40 mg weekly	5.3	0.9	5.1	4.9	8.0	Drug continued, norm. of TET
9	M	30	0.7	80 mg weekly	NA	0.9	0.8	3.1	1.2	Drug continued, norm. of TET
10	M	40	5.8	40 mg weekly	1.6	1.1	4.6	3.1	4.3	Drug continued, norm. of TET
11	M	34	4.9	40 mg weekly	2.0	1.4	8.8	9.5	8.3	Drug continued, norm. of TET
12	M	46	2.9	60 mg weekly	0.9	1.2	4.8	6.3	13.2	Drug continued, norm. of TET
13^a^	M	74	12.8	80 mg weekly	1.3	2.6	11.8	5.6	3.7	Drug continued, norm. of TET
14	M	45	5.8	60 mg weekly	1.3	1.3	6.9	13.7	26.1	Drug withdrawn, norm. of TET
15	F	48	15.4	80 mg weekly	0.8	0.8	1.5	2.9	4.5	Drug continued, norm. of TET
16	F	44	3.4	20 mg weekly	1.0	0.6	0.9	7.5	10.0	Drug continued, norm. of TET Re-exposure to PEGV: caused a 2nd TET period
17	F	54	5.5	60 mg weekly	0.7	0.7	2.7	2.8	4.0	Drug continued, norm. of TET
18	F	41	4.8	60 mg weekly	NA	NA	3.9	NA	4.6	Drug continued, norm. of TET
19	F	61	11.3	160 mg weekly	NA	1.1	4.1	2.4	3.7	Drug continued, norm. of TET
20	F	67	2.2	20 mg weekly	NA	0.9	2.3	2.8	3.7	Drug continued, norm. of TET Re-exposure to PEGV: caused a 2nd TET period
21	F	41	2.5	40 mg weekly	1.3	0.7	1.3	8.4	11.7	Drug continued, norm. of TET
22	F	27	4.1	60 mg weekly	NA	1.0	1.5	4.0	4.8	Drug continued, norm. of TET

Description of TET patients during medical treatment with LA-SSA combined with PEGV

^a^Elevated transaminases due to proven cholecystolithiasis

*LFT* liver function tests, *TET* transient elevated transaminases, *LA-SSA* long-acting somatostatin analogues, *PEGV* pegvisomant, ×*ULN* times upper limit of normal, *Bili* total bilirubin, *Alk. Phos* alkaline phosphatase, *γ*-*GT* *γ*-glutamyltranspeptidase, *AST* aspartate aminotransaminase, *ALT* alanine aminotransferase, *NA* not available

More frequent out-patient clinic visits and, thereby, more frequent assessments of ALT and AST might explain the observed differences in the incidence of TET, as the elevations in transaminases are transient and will pass unnoticed when follow-up intervals are wider. A previously described association between TET and Gilbert’s polymorphism (UGT1A1*28) in a Spanish study (*n* = 36) [58], was not confirmed by the Dutch cohort (*n* = 141) [12].

## Conclusion

Advantages of the combination treatment of LA-SSA and PEGV are high efficacy rates in acromegaly patients, provided that the appropriate PEGV dose is used, which seems to be lower in the combined treatment than during PEGV monotherapy. Tumor shrinkage and cessation of tumor volume was observed in the vast majority of the patients. Disadvantages of combining LA-SSA with PEGV is the economic burden of life-long administration of expensive medicaments and side effects of both drugs can occur. Furthermore, in patients treated with LA-SSA and PEGV, only IGF-I levels can be interpreted as some commercial GH assays are not able to distinguish between GH and PEGV. Although, the combination of LA-SSA and PEGV is generally well tolerated and side effects as lipohypertrophy and elevated transaminase are usually mild and transient, clinical attention towards side effects remain obligate. Therefore, we recommend to frequently rotate the PEGV injection-site as this reduces the risk of (serious) lipohypertrophy. With respect to the elevated transaminases >3 ×ULN, we recommend assessing liver enzymes levels with the same frequency as described by the label of PEGV. Cholelithiasis must be excluded by an ultrasound of the liver and gallbladder. In patients with TET >10 ×ULN, we also recommend performing a liver biopsy and discontinuing PEGV in case of drug-induced hepatitis. PEGV-induced tumor size increase has not been observed to date, but repetitive pituitary imaging remains mandatory.

## Abbreviations

GH: Growth hormone
IGF-I: Insulin-like growth factor I
LA-SSA: Long-acting somatostatin analogue
GHR: Growth hormone receptor
PEGV: Pegvisomant
